# Mode of delivery according to Robson classification and perinatal outcomes in restricted and small for gestational age fetuses

**DOI:** 10.61622/rbgo/2024rbgo30

**Published:** 2024-07-26

**Authors:** Jaqueline Brandão Mazzola, Ana Cristina Perez Zamarian, Ana Carolina Rabachini Caetano, Luiza Grosso Silva Drumond, Vivian Macedo Gomes Marçal, Amanda Botelho, Edward Araujo Júnior, Sue Yasaki Sun, Luciano Marcondes Machado Nardozza

**Affiliations:** 1 Department of Obstetrics Escola Paulista de Medicina Universidade Federal de São Paulo São Paulo SP Brazil Department of Obstetrics, Escola Paulista de Medicina, Universidade Federal de São Paulo, São Paulo, SP, Brazil.

**Keywords:** Fetal growth retardation, Cesarean section, Pregnancy outcome, Infant, small for gestational age, Gestational age, Infant, newborn, Fetus, Robson classification

## Abstract

**Objective:**

To evaluate the mode of delivery according to Robson classification (RC) and the perinatal outcomes in fetal growth restriction (FGR) and small for gestational age (SGA) fetuses.

**Methods:**

Retrospective cohort study by analyzing medical records of singleton pregnancies from two consecutive years (2018 and 2019). FGR was defined according to Delphi Consensus. The Robson groups were divided into two intervals (1–5.1 and 5.2–10).

**Results:**

Total of 852 cases were included: FGR (n = 85), SGA (n = 20) and control (n=747). FGR showed higher percentages of newborns < 1,500 grams (p<0.001) and higher overall cesarean section (CS) rates (p<0.001). FGR had the highest rates of neonatal resuscitation and neonatal intensive care unit admission (p<0.001). SGA and control presented higher percentage of patients classified in 1 - 5.1 RC groups, while FGR had higher percentage in 5.2 - 10 RC groups (p<0.001). FGR, SGA and control did not differ in the mode of delivery in the 1-5.1 RC groups as all groups showed a higher percentage of vaginal deliveries (p=0.476).

**Conclusion:**

Fetuses with FGR had higher CS rates and worse perinatal outcomes than SGA and control fetuses. Most FGR fetuses were delivered by cesarean section and were allocated in 5.2 to 10 RC groups, while most SGA and control fetuses were allocated in 1 to 5.1 RC groups. Vaginal delivery occurred in nearly 60% of FGR allocated in 1-5.1 RC groups without a significant increase in perinatal morbidity. Therefore, the vaginal route should be considered in FGR fetuses.

## Introduction

Fetal growth restriction (FGR) is a common obstetric complication, affecting 5%–10% of singleton pregnancies and is associated with adverse perinatal outcomes such as fetal and neonatal death, prematurity and need for neonatal respiratory support.^(1)^The most appropriate way to define FGR would be the fetus that has not reached its intrauterine growth potential due to some degree of placental insufficiency.^(2)^However, there is no consensus regarding on objective diagnostic criteria,^(3)^ considering that some fetuses are constitutionally smaller due to the influence of genetics and ethnicity.^(4)^These fetuses are referred to as small for gestational age (SGA) and exhibits similar morbidity and mortality rates to appropriate for gestational age (AGA) fetuses.

Robson classification (RC), published in 2001,^(5)^allows allocation of parturients in ten groups based on six parameters [parity, gestational age (GA) at delivery, previous cesarean section, number of fetuses, fetal presentation, and labor onset]. It is simple, objective, uses well-established obstetric criteria and is completely inclusive and mutually exclusive, so that each patient can only be allocated in one of the groups. This fact, in addition to enabling further reliable comparisons, facilitates the identification of the characteristics of pregnant women that progress to cesarean deliveries, and detects modifiable factors in order to reduce cesarean rates. Chart 1 describes the characteristics of parturients included in each group. Since 2015, the World Health Organization (WHO) has been recommended RC to monitor cesarean section rates in maternity hospitals.^(6)^

**Chart 1:** Robson classification

	Parity	Gestational age	Previous cesarean section	Number of fetuses	Fetal presentation	Onset of labor
1	Nulliparous	≥ 37 weeks	No	Single	Cephalic	Spontaneous
2a	Nulliparous	≥ 37 weeks	No	Single	Cephalic	Induced
2b	Nulliparous	≥ 37 weeks	No	Single	Cephalic	Pre-labor CS
3	Multiparous	≥ 37 weeks	No	Single	Cephalic	Spontaneous
4a	Multiparous	≥ 37 weeks	No	Single	Cephalic	Induced
4b	Multiparous	≥ 37 weeks	No	Single	Cephalic	Pre-labor CS
5.1	Multiparous	≥ 37 weeks	1	Single	Cephalic	-
5.2	Multiparous	≥ 37 weeks	≥2	Single	Cephalic	-
6	Nulliparous	-	-	Single	Breech	-
7	Multiparous	-	-	Single	Breech	-
8	-	-	-	Multiple	-	-
9	-	-	-	Single	Transverse	-
10	-	< 37 weeks	-	Single	Cephalic	-

CS - cesarean section

The objective of this study was to evaluate the mode of birth delivery, according to Robson classification, and the perinatal outcomes in FGR and SGA, comparing them with other fetus deliveries of the maternity hospital with estimated weight above the tenth percentile.

## Methods

A retrospective cohort study was conducted by analyzing medical records at São Paulo Hospital of the Federal University of São Paulo - Paulista School of São Paulo (UNIFESP - EPM), from January 2018 to December 2019. Given the design and objectives of the study, it was important to include all eligible patients to avoid sampling bias. If it was the possibility of contact with the participants who remained at our service, a consent form was written and available. For participants who could not be contacted, a declaration of confidentiality and data anonymization was signed, in accordance with local recommendations.

The exclusion criteria were: multiple gestation, fetal malformation, or fetal infection suspected on ultrasound or identified in the postnatal period. This study classified FGR according to the definition published in 2016, using the Delphi procedure that consisted to the following: Early FGR (< 32 weeks) - three solitary parameters are described: abdominal circumference (AC) < 3rd centile, estimated fetal weight (EFW) < 3rd centile or absent end-diastolic flow in the umbilical artery (UA) Doppler. Contributory parameters are: AC or EFW < 10th centile combined with a pulsatility index (PI) > 95th centile in either the UA or in the uterine artery Doppler. Late FGR (≥ 32 weeks) - two solitary parameters (AC or EFW < 3rd centile) and the presence of at least two contributory parameters (EFW or AC < 10th centile, AC or EFW crossing centiles > two quartiles on growth charts, cerebroplacental ratio <5th centile or UA-PI > 95th centile) were defined.^(7)^

The patients were classified into three groups: (I) FGR – EFW < 10th percentile by the Hadlock et al.^(8)^table and met FGR criteria, according to Delphi Consensus;^(7)^(II) SGA – EFW < 10th percentile by the Hadlock et al.^(8)^table without the FGR criteria, according to Delphi Consensus;^(7)^(III) Control – other deliveries of the maternity hospital with EFW > 10th percentile, according to the Hadlock et al.^(8)^table.

For the definition of FGR or SGA, the last ultrasound scan report performed in the service was evaluated. In the reports, the Doppler parameters of the umbilical and middle cerebral arteries were evaluated according to the Arduini and Rizzo^(9)^ curve and the uterine arteries Doppler were evaluated according to the Gómez et al.^(10)^ curve.

Management of FGR is summarized in chart 2 according to the local protocol at the time of delivery.^(11)^The adverse perinatal outcomes evaluated were: birth weight, 5-min Apgar score, umbilical cord pH, need for neonatal resuscitation, neonatal intensive care unit (NICU) admission, fetal death, and in-hospital death.

**Chart 2:** Local management of fetal growth restriction

Stage	Description	Viability monitoring	Birth
SGA	3rd > EFW/AC < 10th Normal Doppler	Monitor vitality every to 2 weeks	Expectant management Labor induction at 40 weeks
FGR I	EFW/AC < 3rd Normal Doppler	Monitor vitality every to 2 weeks until 34 weeks and every week after 34 weeks	Expectant management Labor induction between 37 and 38 weeks
FGR II	Abnormalities in UA, MCA or CPR	Monitor vitality twice a week	Expectant management Labor induction at 37 weeks
FGR III	Absent-end diastolic in UA	Hospitalization and daily monitoring	Expectant management Birth at 34 weeks by elective cesarean
FGR IV	UA with reversed-end diastolic or DV PI> 95^th^	Hospitalization and daily monitoring	Delivery when viability (26 – 28 weeks) by elective cesarean section
FGR V	Reversed a-wave DV or frequent FHR decelerations		

SGA - small for gestational age; EFW - estimated fetal weight, AC - abdominal circumference; UA - umbilical artery; MCA - medial cerebral artery; CPR - cerebroplacental ratio; PI - pulsatility index; DV - ductus venosus; FHR - fetal heart rate

To perform the comparison of the three groups regarding the mode of delivery by RC, each group was aggregated into two intervals, as shown below: (I) Robson Group 1 – 5.1 = all singleton pregnancies of a fetus in cephalic presentation regardless of parity and onset of labor (spontaneous, induced, or absent), admitted with GA ≥ 37 weeks and having up to one previous cesarean section; (II) Robson Group 5.2–10 = all singleton pregnancies ≥ 37 weeks with two or more previous cesarean sections, cephalic preterms, and all non-cephalic presentations regardless of GA. It should be noted that Group 8 (multiple gestation) was not included in our evaluation. The choice of intervals was based on the fact that the Robson Group (1 -5.1) corresponded to patients with a higher probability of vaginal delivery, both due to the intrinsic characteristics of the pregnant women in this group and to our follow-up management protocol.

We classified the indications for cesarean section into three groups. (I) Fetal indication – indication by any means of objective evaluation of fetal vitality such as Doppler abnormalities on ultrasound or fetal heart rate on intermittent auscultation or on cardiotocography. (II) Obstetric indication – any antepartum or intrapartum indication for conditions predominantly related to the gestational period or intrapartum such as arrested cervical dilation, arrested progression of fetal presentation and prior uterine scars. (III) Maternal indication – any maternal medical conditions induced by pregnancy or previously present comorbidity that worsens during pregnancy or contraindicates the vaginal delivery.

Data were tabulated in Excel 2010 spreadsheet (Microsoft Corp., Redmond, WA, USA) and analyzed using IBM SPSS Statistics for Windows software (version 22.0, IBM Corp, Armonk, NY, USA). The information on quantitative variables was summarized using mean, standard deviation (SD), median, interquartile range (IQR), and minimum and maximum values. The information from the qualitative variables was summarized using simple frequency and percentage.

To compare the groups in relation to quantitative variables, which did not show normal distribution, the Kruskal–Wallis non-parametric test was used, followed by the Mann–Whitney non-parametric test with Bonferroni correction. To compare the groups with regard to the qualitative variables, the Chi-Square test or, when necessary, the Likelihood ratio test was used. In all analyses, p-value <0.05 was considered significant.

This study was approved by the Research Ethics Committee of UNIFESP (CAAE: 29823419.0.0000.5505) (CEP: 4.053.519).

## Results

During the study period, 1063 pregnant women were admitted, 123 being fetuses with EFW < 10th percentile and 940 with EFW > 10th percentile (control group). Of the 123 fetuses with EFW < 10th percentile, 18 were excluded: 3 congenital heart diseases, 1 ambiguous genitalia, 10 genetic alterations (3 trisomy 21, 1 monosomy X, and 6 undefined genetic syndromes), 1 bilateral congenital cataract, 1 cytomegalovirus, and 2 skeletal dysplasias. Of the 105 fetuses included, 85 were classified as FGR and 20 as SGA. Of the 940 cases in the control group, 193 fetuses were excluded: 45 multiple pregnancies, 120 fetal malformations, 19 fetal infections, 6 out-of-service deliveries, and 3 cases with incomplete data in the medical records.

Table 1 presents the sociodemographic maternal characteristics and adverse perinatal outcomes in the three groups evaluated. The mean maternal age ± SD in the control, SGA, and FGR groups were 30.4 ± 7.1, 27.9 ± 6.4, and 28.3 ± 7.5 years, respectively, being significantly lower in the FGR group compared to the control group (p=0.012).

**Table 1:** Sociodemographic maternal characteristics and adverse perinatal outcomes according to the three groups evaluated

Variables	FGR (n=85) n(%)	SGA (n=20) n(%)	Control (n=747) n(%)	p-value
Maternal age (years)				
Mean (Standard Deviation)	28.3(7.5)	27.9(6.4)	30.4(7.1)	0.012^#^
Median (Percentile 25–75)	28(22 – 34)	29(21 – 33)	30(25 – 36)	
Minimum–Maximum	(14 – 45)	(18 – 37)	(14 – 45)	
Education				
ILL + IEE	6(7.0)	1(5.0)	66(8.8)	0.317^*^
CEE + IHS	7(8.2)	3(15.0)	119(15.9)	
CHS + IHE	53(62.4)	11(55.0)	400(53.6)	
CHE	14(16.5)	5(25.0)	110(14.7)	
Ignored	5(5.9)	0(0)	52(7.0)	
Race				
Asian	1(1.2)	0(0)	6(0.8)	0.511^*^
White	41(48.2)	13(65.0)	348(46.6)	
Mixed	7(8.2)	3(15.0)	88(11.8)	
Black	36(42.4)	4(20.0)	298(39.9)	
Ignored	0(0)	0(0)	7(0.9)	
Mode of delivery				
Cesarean section	58(68.2)	7(35.0)	352(47.1)	< 0.001^**^
Vaginal	27(31.8)	13(65.0)	395(52.9)	
Gestational age at delivery				
<28 weeks	7(8.3)	0(0)	18(2.4)	< 0.001^*^
28–30 weeks	9(10.6)	0(0)	17(2.3)	
31–33 weeks	11(12.9)	0(0)	16(2.1)	
34–36 weeks	16(18.8)	1(5.0)	95(12.7)	
≥37 weeks	42(49.4)	19(95.0)	601(80.5)	
Birth weight				
< 500 g	5(5.9)	0(0)	7(0.9)	< 0.001^*^
500 – 999 g	10(11.8)	0(0)	12(1.6)	
1000 - 1499g	9(10.6)	0(0)	15(2.0)	
1500 - 2499 g	43(50.6)	10(50.0)	63(8.5)	
≥ 2500g	18(21.1)	10(50.0)	650(87.0)	
5-min Apgar score				
Mean (Standard Deviation)	9(1.3)	9,2(0.9)	9,4(1.1)	0.001^#^
Median (Percentile 25–75)	9(9–10)	9(9–10)	10(9–10)	
Minimum–Maximum	(3–10)	(7–10)	(0–10)	
< 7	5(6.0)	1(5.3)	17(2.3)	0.184*
≥7	78(94.0)	18(94.7)	715(97.7)	
Umbilical cord pH				
Mean (Standard Deviation)	7.2(0.1)	7,2(0.1)	7,2(0.1)	0.253^#^
Median (Percentile 25–75)	7.2(7.2 – 7.3)	7.2(7.1 – 7.3)	7.3(7.2 – 7.3)	
Minimum–Maximum	(7.0 – 7.4)	(7.0 – 7.3)	(6.7 – 7.5)	
Neonatal resuscitation				
No	62(74.7)	16(84.2)	664(90.7)	< 0.001^**^
Yes	21(25.3)	3(15.8)	68(9.3)	
NICU admission				
No	18(21.7)	10(52.6)	484(66.1)	< 0.001^**^
Yes	65(78.3)	9(47.4)	248(33.9)	
Fetal death				
No	83(97.6)	19(95.0)	732(98.0)	0.723^*^
Yes	2(2.4)	1(5.0)	15(2.0)	
In-hospital death				
No	80(96.4)	19(100)	724(98.9)	0.213^*^
Yes	3(3.6)	0(0)	8(1.1)	

FGR - fetal growth restriction; SGA - small for gestational age; ILL - illiterate; IEE - incomplete elementary education; CEE -complete elementary education; IHS - incomplete high school; CHS - complete high school; IHE - incomplete higher education; CHE - complete higher education; NICU - neonatal intensive care unit; #Kruskal–Wallis; *Likelihood ratio; **Chi-square

We observed higher cesarean section rates in FGR compared to SGA and control group (p < 0.001). The percentage of GA at birth ≥ 37 weeks was significantly higher in the SGA and control than in the FGR group (p < 0.001). In addition, the median GA at delivery was significantly lower in FGR than in SGA and control groups (p < 0.001), which did not differ from each other with respect to this variable. The control group had a higher percentage of newborn (NB) weight ≥ 2500 grams, half of the SGA had NB group had weight ≥ 2500 grams, and the other half had NB weight in the 1500–2499 grams range. FGR was the only group that showed a higher percentage of NBs with weights < 1500 grams, when compared to the other groups (p < 0.001). The median 5-min Apgar score was significantly higher in the control group than in the other groups (p < 0.001), which did not differ from one another. However, when we categorized the Apgar score into the clinically significant value (< 7 and ≥ 7) we found no difference between the groups (p=0.184). The percentage of neonatal resuscitation was significantly higher in the FGR group than the control group (p < 0.001). The percentage of NICU admission was significantly higher in FGR group than in the other groups (p < 0.001).

Table 2 presents the variables of Robson classification according to the three groups evaluated. The percentage of nulliparous women was significantly lower in the control group than in the others (p < 0.001), which did not differ from each other with respect to parity. The percentage of previous cesarean section was significantly lower in FGR than in SGA and control (p < 0.001), which did not differ from one another with respect to this variable. The percentage of breech presentation was significantly higher in the FGR group than in the other groups (p < 0.001), which did not differ from one another with respect to this variable. The percentage of absent labor was significantly higher in the FGR (p< 0.001). The percentage of spontaneous labor was significantly higher in the control group, while the percentage of induced labor was significantly higher in the SGA group (p < 0.001).

**Table 2:** Robson classification variables according to the three groups evaluated

Robson classification variables	FGR (n=85) n(%)	SGA (n=20) n(%)	Control (n=747) n(%)	p-value
Parity				< 0.001^**^
Multiparity	31(36.5)	9(45.0)	464(62.1)	
Nulliparity	54(63.5)	11(55.0)	283(37.9)	
Previous cesarean section				0.027^**^
No	70(82.4)	13(65.0)	517(69.2)	
Yes	15(17.6)	7(35.0)	230(30.8)	
Fetal presentation				0.001^*^
Cephalic	70(82.4)	20(100)	702(93.9)	
Breech	15(17.6)	0(0)	40(5.4)	
Transverse	0(0)	0(0)	5(0.7)	
Onset of labor				< 0.001^**^
Absent	51(60.0)	7(35.0)	223(29.9)	
Spontaneous	3(3.5)	3(15.0)	278(37.2)	
Induced	31(36.5)	10(50.0)	246(32.9)	

FGR - fetal growth restriction; SGA - small for gestational age; (I) multiple gestation was not included in our study; (II) gestational age at delivery is described in table 1; *Likelihood ratio; **Chi-square

Regarding cesarean section indications, fetal and obstetric indications were respectively higher and lower in the FGR group (p < 0.001). Maternal indication for cesarean section was significantly higher (p < 0.001) in the SGA group (Table 3).

**Table 3:** Comparison between the three groups regarding indications for cesarean section

Comparison	FGR n(%)	SGA n(%)	Control n(%)	p-value
Indications for cesarean section				< 0.001^*^
Fetal	31(53.4)	0(0)	65(18.5)	
Obstetric	16(27.6)	4(57.1)	229(65.1)	
Maternal	11(19.0)	3(42.9)	58(16.4)	
Total	58(100)	7(100)	352(100)	

FGR - fetal growth restriction; SGA - small for gestational age; *Likelihood ratio

There was a significant difference between the mode of delivery in relation to Robson groups. The percentage of vaginal deliveries was significantly higher in the interval of Robson groups 1 to 5.1, than in the interval 5.2 to 10 (p < 0.001). In the SGA and control groups there was a higher percentage of pregnant women in the range 1–5.1, while in the FGR group there was a higher percentage in the interval 5.2–10 (p < 0.001). Table 4 presents the comparison between the three groups regarding the mode of delivery for each of Robson’s group intervals. For Robson interval 1–5.1, no significant difference was observed between the groups regarding the mode of delivery; as all groups have a higher percentage of vaginal deliveries (p = 0.476). For Robson groups 5.2–10, a higher percentage of cesarean sections was observed in all groups, but the percentage of pregnant women in the control group who progressed to vaginal delivery in this interval of RC was higher than in the other groups (p=0.009). The distribution of births according to the 10 Robson groups report is available in chart 3.

**Table 4:** Comparison between the three groups regarding the type of delivery for each of Robson’s group intervals

Comparison	Cesarean section n(%)	Vaginal delivery n(%)	p-value
Robson Group 1 to 5.1			0.476^**^
FGR	15(40.5)	22(59.5)	
SGA	4(23.5)	13(76.5)	
Control	188(36.2)	331(63.8)	
Total	207(36.1)	366(63.9)	
Robson Group 5.2 to 10			0.009^*^
FGR	43(89.6)	5(10.4)	
SGA	3(100)	0(0)	
Control	164(71.9)	64(28.1)	
Total	210(75.3)	69(24.7)	

FGR - fetal growth restriction; SGA - small for gestational age; *Likelihood ratio; **Chi-square

**Chart 3:** Distribution of births according to the 10 Robson groups report

Group	Vaginal Delivery in Group				Number of CS in Group				Number of Women in Group				Group Size (%)				Group CS Rate (%)				Absolute Group Contribution to Overall CS Rate (%)				Relative Contribution of Group to Overall CS Rate (%)			
	T	FGR	SGA	C	T	FGR	SGA	C	T	FGR	SGA	C	T	FGR	SGA	C	T	FGR	SGA	C	T	FGR	SGA	C	T	FGR	SGA	C
1	80	2	1	77	16	0	0	16	96	2	1	93	11.3	0.2	0,1	11	16.7	0	0	16.7	1,9	0	0	1.9	3.8	0	0	3.8
2a	80	13	9	58	63	9	1	53	143	22	10	111	16.8	2.6	1.2	13.0	44.1	6.3	0.7	37.1	7,4	1.1	0.1	6.2	15.1	2.2	0.2	12.7
2b	0	0	0	0	19	2	0	17	19	2	0	17	2.2	0.2	0.0	2.0	100	10.5	0.0	89.5	2,2	0.2	0.0	2.0	4.6	0.5	0.0	4.1
3	83	0	1	82	5	0	0	5	88	0	1	87	10.3	0.0	0.1	10.2	5.7	0.0	0.0	5.7	0,6	0.0	0.0	0,6	1.2	0.0	0.0	1.2
4a	70	3	1	66	29	2	0	27	99	5	1	93	11.6	0.6	0.1	10.9	29.3	2.0	0.0	27.3	3,4	0.2	0.0	3.2	7.0	0.5	0.0	6.5
4b	0	0	0	0	3	0	0	3	3	0	0	3	0.4	0.0	0.0	0.4	100	0.0	0.0	100	0,4	0.0	0,0	0.4	0.7	0.0	0.0	0.7
5.1	53	4	1	48	72	2	3	67	125	6	4	115	14.7	0.7	0.5	13.5	57.6	1.6	2.4	53.6	8,5	0.2	0.4	7.9	17.3	0.5	0.7	16.1
5.2	0	0	0	0	63	0	2	61	63	0	2	61	7.4	0	0.2	7.2	100	0	3.2	96.8	7,4	0	0.2	7.2	15.1	0	0.5	14.6
6	5	0	0	5	14	7	0	7	19	7	0	12	2.2	0.8	0	1.4	73.6	36.8	0	36.8	1,6	0.8	0	0.8	3.4	1.7	0	1.7
7	2	0	0	2	34	8	0	26	36	8	0	28	4.2	0.9	0	3.3	94.4	22.2	0	72.2	3,9	0.9	0	3	8.1	1.9	0	6.2
9	0	0	0	0	5	0	0	5	5	0	0	5	0.6	0	0	0.6	100	0	0	100	0,6	0	0	0.6	1.2	0	0	1.2
10	62	5	0	57	94	28	1	65	156	33	1	122	18.3	3.9	0.1	14.3	60.2	17.9	0.6	41.7	11	3.3	0.1	7.6	22.5	6.7	0.2	15.6
X	0				0				0				-				-				-				-			
Total	435	27	13	395	417	58	7	352	852	85	20	747	100				-				-				100			

% - percentage; CS - cesarean section; T - total; FGR - fetal growth restriction; SGA - small for gestational age; C - control; X - unclassifiable cases

## Discussion

Our study found a higher percentage of fetuses classified as FGR than SGA, unlike what is described in literature.^(12)^This higher proportion can be explained by the complexity of the cases followed up and referred to our service, which is a tertiary center that treats pregnant women with several comorbidities. Such comorbidities have the potential to generate severe and early placental insufficiency, with a greater likelihood of FGR.

Placental implantation abnormalities are very common in the first pregnancy,^(13)^which is consistent with our findings of lower median age in the FGR group and a higher percentage of nulliparous women in pregnancies with estimated fetal weight < 10th percentile. Cesarean section was statistically more frequent in the FGR group, as expected, since these fetuses have a higher risk of Doppler abnormalities and a higher risk of intrapartum fetal vitality compromise. A retrospective study of 100 cases of labor induction in fetuses with late FGR showed 63% vaginal deliveries, 32% cesarean sections, and 5% operative vaginal deliveries. Doppler abnormalities of the AU, middle cerebral artery and cerebroplacental ratio were observed to be associated with increased risk of cesarean section.^(14)^As expected, GA at delivery was low in the FGR group. Deliveries ≥ 37 weeks occurred in 49.4% in the FGR, 95% in the SGA, and 80.5% in the control groups. A Spanish study published in 2022 involving 655 AGA (appropriate for gestational age), 62 SGA, 132 early FGR, and 57 late FGR fetuses (classified by Delphi Consensus) found similar GA at delivery in AGA (39: interquartile range - IQR 39–40) and SGA (39: IQR 38–40) fetuses and statistically lower in fetuses classified as early FGR (32: IQR 29–38) when compared to the other groups. A difference was observed in the GA of fetuses with late FGR (38: IQR 36–49) when compared to AGA and SGA.^(15)^

The need for neonatal resuscitation and NICU admission were significantly higher in the FGR group. In a retrospective longitudinal study, pregnant women carrying fetuses with early FGR, late FGR, SGA, and AGA were included for correlation with adverse neonatal outcomes. Logistic regression model including FGR type and GA at delivery was considered in predicting risk of NICU admission. Model including only the type of FGR (early or late) better predicted risk of need for neonatal resuscitation, respiratory distress, and birth at GA < 32, 34, and 37 weeks, respectively.^(16)^

In the evaluation of all the deliveries included in the study, most of the pregnant women who progressed to vaginal delivery were classified in Robson groups 1 to 5.1. When we compared the distribution of the pregnant women in the two intervals of Robson groups, there was a higher percentage in the FGR group in the interval 5.2–10. This probably occurred because of a higher incidence of preterm births with cephalic presentation and a higher incidence of breech presentation associated with prematurity in fetuses with a high probability of true growth restriction and a high probability of being born prematurely.

Regarding the type of delivery, there was no statistical difference between the groups in Robson intervals 1–5.1, with vaginal delivery being the most common, which is in line with Robson classification that aims to screen pregnant women with a high probability of vaginal delivery. In the second interval (Robson 5.2–10), cesarean section was more common in all groups.

Analyzing only the FGR group it was intuitive to find a high overall percentage of cesarean deliveries due to the potential severity of the cases. However, when we selected the most eligible patients for vaginal delivery in the FGR group (Robson 1–5.1), the percentage of vaginal delivery was 59.5% and no increase in stillbirth was observed when compared to the other groups.

To date, there are no studies specifically evaluating the contribution of pregnant women with EFW < 10th percentile on cesarean section rates based on Robson classification. This evaluation is important because the evidences show that vaginal delivery, in selected FGR fetuses and with adequate monitoring, may be possible and safe for the mother–fetus binomial.

In 2016, a study of cesarean section rates in Brazil was published by Nakamura-Pereira et al.,^(17)^using data from 2011 to 2012 from the public and private facilities. The sample included 23,940 women, who were classified into one of Robson’s ten groups. The total cesarean section rate was 51.9% (42.9% in the public sector and 87.9% in the private sector), with 70% performed in pregnant women of the Groups 2, 5, and 10. High-risk pregnant women had significantly higher rates of cesarean sections than low-risk pregnant women in almost all groups in the public sector. Group 2 was the largest in this study, corresponding to 20% of the entire population, and 70% of the pregnant women in this group underwent antepartum cesarean section and 30% had induced labor.

Similar data was found in our study regarding the overall cesarean section rate (48.9%) and the groups that contributed the most were Groups 2 (19.7%), 5 (32.4%) and 10 (22.5%) totaling 74.6% of the total cesarean sections. However, in our study, the largest group was Group 10, corresponding to 18.3% of the entire population, probably secondary to the characteristics of the maternity hospital, which is a tertiary referral center for high-risk cases.

This study had some limitations, some of which are inherent related to retrospective studies, such as the use of pre-existing data not systematically collected for this specific purpose. The results reflect more outcomes in the FGR group than in the SGA group, due to the relatively small number of participants in the SGA group (n=20) and therefore the comparison of the SGA group with the others may not be accurate. Our control group did not represent the usual low-risk obstetric population, as most of our patients already have some high-risk condition.

Selection bias was mitigated by the fact that all data collection and tabulation was carried out by a single researcher, who used all means available in the electronic medical record platform to locate the medical records of the pregnant women and their newborns. The medical records of all patients in the RCF and SGA groups were found. In the control group, only three records of the 940 eligible patients could not be found, therefore, resulting in less than 0.5% of loss of information for this reason.

## Conclusion

Fetuses with FGR had higher cesarean section rates and worse perinatal outcomes than SGA and AGA fetuses. However, when we evaluated mortality data (fetal death and in-hospital death) no statistical difference was observed in our study population, which can be attributed to a good prenatal care follow-up of these cases. Fetuses with FGR had higher cesarean section rates in groups 5.2–10. On the other hand, when patients eligible for vaginal delivery in the FGR group (Robson 1–5.1) were selected, there were approximately 60% vaginal deliveries with satisfactory perinatal outcomes. This data is important to motivate the professionals about the safety in trying and conducting labor in this context in eligible patients.
